# First report of human infection with *Leptospira interrogans* serovar Bratislava in the Eastern Black Sea region of Turkey

**DOI:** 10.1590/S1678-9946202365048

**Published:** 2023-09-08

**Authors:** Ersin Kuloglu, Kubilay Issever

**Affiliations:** 1 Giresun University, Faculty of Medicine, Department of Internal Medicine, Giresun, Turkey

**Keywords:** Leptospirosis, Fever, Rash, Acute renal failure

## Abstract

Leptospirosis is one of the most common zoonotic bacterial infections worldwide. It is an infection that usually affects people with low socioeconomic status, with morbidity and mortality risk. The clinical course of the disease may range from mild, featuring nonspecific clinical signs and symptoms, to severe, resulting in death. The respective studies conducted in Turkey indicate that leptospirosis seropositivity in animals and humans is higher in coastal and rural areas. Turkey’s Eastern Black Sea Region has a humid climate with heavy rainfalls and a large population of mice and other rodents. However, a *Leptospira interrogans* serovar Bratislava case is yet to be reported in this region. This article reports the case of a 38-year-old patient who presented fever and acute renal failure and was diagnosed with *Leptospira interrogans* serovar Bratislava after hospitalization.

## INTRODUCTION

Leptospirosis usually affects people with low socioeconomic status and is estimated to occur in approximately one million cases annually, of which 58,900 result in death. The mortality rate in leptospirosis cases has been reported as 6.85%^1^. Leptospirosis disease was first described by Adolph Weil in 1886 as a febrile disease observed in people who come into contact with open-air waters and progresses with icterus, fever, splenomegaly, renal failure, and conjunctivitis. For this reason, the severe form of leptospirosis is also called Weil’s disease^2^. Leptospirosis is a disease of primarily wild and domestic mammals caused by the pathogenic *Leptospira* species. Transmission to humans generally occurs through direct contact with the urine or any tissue of infected animals, which can remain carriers for a long time, or indirectly through contaminated water, soil, and vegetables^1,3^. Although mice are the most common reservoirs of spirochetes, livestock, dogs, wild mammals, and cats can also carry them^3^. Many serovars of *Leptospira interrogans* (*L. interrogans*) can infect humans. Epidemiologic studies were mainly conducted on animals rather than humans in the literature; thus, knowledge about the prevalence of *Leptospira* serovars worldwide is limited. The clinical presentation of leptospirosis ranges from mild, which comprises self-limiting acute febrile illness, to serious complications such as myocardial involvement, hepatic derangements, pulmonary hemorrhage, and acute kidney injury^3^. Isolation of the causative agent is required for definitive diagnosis; however, due to the difficulties in isolation and long durations of culture test results, the leptospirosis diagnosis is usually made with serological tests. The most commonly used serological test is the microscopic agglutination test (MAT)^4^. This is the report of a case from a region where *L. interrogans* serovar Bratislava (LISB) had never been reported before.

## CASE REPORT

The 38-year-old female patient, who had no known systemic disease other than lumbar disc herniation and cervical disc herniation, sought medical care at a state hospital in the Eastern Black Sea region of Turkey with complaints of weakness, widespread muscle pain, unmeasured fever, night sweats, loss of appetite, and redness in the face and neck region for about a week. The laboratory examinations revealed that the patient had elevated acute phase reactants and renal function tests. The patient was then referred to the Giresun University Training and Research Hospital with a preliminary diagnosis of vasculitis. The patient’s vital signs measured in the hospital’s emergency department were as follows: arterial blood pressure: 100/60 mmHg, oxygen saturation (sO2): 95%, pulse in sinus rhythm: 100/min, and temperature: 36.7 °C. The physical examination found that her general condition was moderate, and she was conscious, cooperative, and oriented. There was a V-shaped erythematous rash accompanied by increased temperature in the neck and the buccal region of the face bilaterally. The patient’s lung sounds were normal. No tenderness or defense-rebound positivity was observed with palpation of the abdomen. There was also no bilateral pretibial edema.

The patient’s lab parameters at the time of admission are shown in Table 1. These findings revealed a mild metabolic acidosis along with mild neutrophil leukocytosis, normocytic anemia, severely increased acute phase reactants such as erythrocyte sedimentation rate (ESR), C-reactive protein (CRP), and ferritin, moderately increased creatinine, and decreased B12 levels. Other lab test results were within normal range, such as INR, TSH, urinalysis, liver enzymes, electrolytes, and ELISA tests.

**Table 1:** Laboratory test results of the patients at admission.

Parameter	Admıssıon	Reference interval
pH	7.33	7.35 - 7.45
PO_2_	27	83 - 108 mmHg
SO_2_	44	% 95 - 99
lactate	1.4	0.5 -1.6 mmol/L
HCO_3_	21	22- 26 mmol/L
PCO_2_	42	35- 48 mmHg
WBC (white blood cell count)	13,200	4,000- 11,000/mm^3^
Neutrophil	12,240	2,000 -7,000/mm^3^
Lymphocyte	610	800 - 4,000/mm^3^
HGB (hemoglobin)	10.5	12 – 16 g/dL
MCV (mean corpuscular volume)	88.8	80 – 96 fL
Platelet	238,000	150,000 – 450,000/mm^3^
ESR (erythrocyte sedimentation rate)	99	0 – 20 mm/hour
Glucose	140	74 – 100 mg/dL
Creatinine	3.4	0.5 – 0.9 mg/dL
ALT (alanine aminotransferase)	29	0 – 33 U/L
AST (aspartate aminotransferase)	32	0 – 32 U/L
Total bilirubin	0.2	0 – 1.2 mg/dL
Direct bilirubin	0.1	0 – 0.3 mg/dL
LDH (lactate dehydrogenase)	206	135 – 214 u/L
Amylase	60	28 – 100 u/L
ALP (alkaline phosphatase)	117	0 – 145 mg/dL
γGT (gama glutamyl transferase)	82	5- 36 u/L
Calcium	8.9	8.6 – 10.2 mg/dL
Sodium	138	136 – 145 mmol/L
Potassium	3.8	3.5 – 5.1 mmol/L
CRP (c-reactive protein)	360	0 – 5 mg/L
PT (prothrombin time)	15.1	8.4 – 10.6 seconds
APTT (activated partial thromboplastin time)	30.0	23.6 – 30.6 seconds
INR (international normalized ratio)	1.0	0.8 – 1.2
HBsAg (hepatitis b surface antigen)	(-)	-
Anti-HBs (hepatitis b surface antibodies)	(-)	-
Anti-HCV (hepatitis c virüs antibodies)	(-)	-
Anti-HIV (human immunodeficiency vrius antibodies)	(-)	-
TSH (thyroid stimulating hormone)	1.1	0.5 – 4.3 mu/L
FT4 (free thyroxine)	1.0	0.7 – 1.8 ng/dL
Vitamin B12	142	187 – 833 pg/ml
Ferritin	1,034	15 – 150 ng/ml
HbA1c (hemoglobin A1c)	5.8	3.5 – 5.6 %
Complete urine analysis; pH	5.0	
Leukocytes	8	0-5
Erythrocytes	4	0-5
Squamous epithelial cells	23	0-5
Bacteria	0	0-5
Ketone	(-)	-
Leukocyte esterase	(-)	-
Nitrite	(-)	-
Antinuclear antibody (ANA)	(-)	-
Complement component 3 (C3)	123	90-180 mg/dL
Complement component 4 (C4)	39	16-48 mg/dL
Rheumatoid factor (RF)	6	0-14 IU/ml
Anti-citric citrullinated peptide (Anti-CCP) antibody	<7	0-17 IU/ml
Cytoplasmic anti-neutrophil cytoplasmic antibody (c-ANCA)	4.6 (-)	0-20 unit
Perinuclear anti-neutrophil cytoplasmic antibody (p-ANCA)	3.4 (-)	0-20 unit
Immunoglobulin G (IgG)	9	7-16 g/L
Immunoglobulin G (IgA)	1.3	0.7-4 g/L
Immunoglobulin G (IgM)	2.4	0.4-2.3 g/L
Human leukocyte antigen B27 (HLA-B27)	(-)	-

The posteroanterior chest X-ray and non-contrast abdominal tomography performed in the emergency department did not indicate any pathological finding that could explain the patient’s clinical presentation. The patient was then hospitalized for differential diagnosis and treatment in the internal medicine service. The patient was prescribed isotonic fluid therapy for the possible prerenal cause of renal damage and 2 g/day IV ceftriaxone therapy for the potential bacterial infection. Infectious disease consultation revealed a recommendation for rheumatology consultation, suggesting that the possible underlying cause was considered rheumatologic rather than infectious. However, the rheumatological workup revealed no sign of vasculitic or auto-inflammatory disease (Table 1).

**Figure 1:**
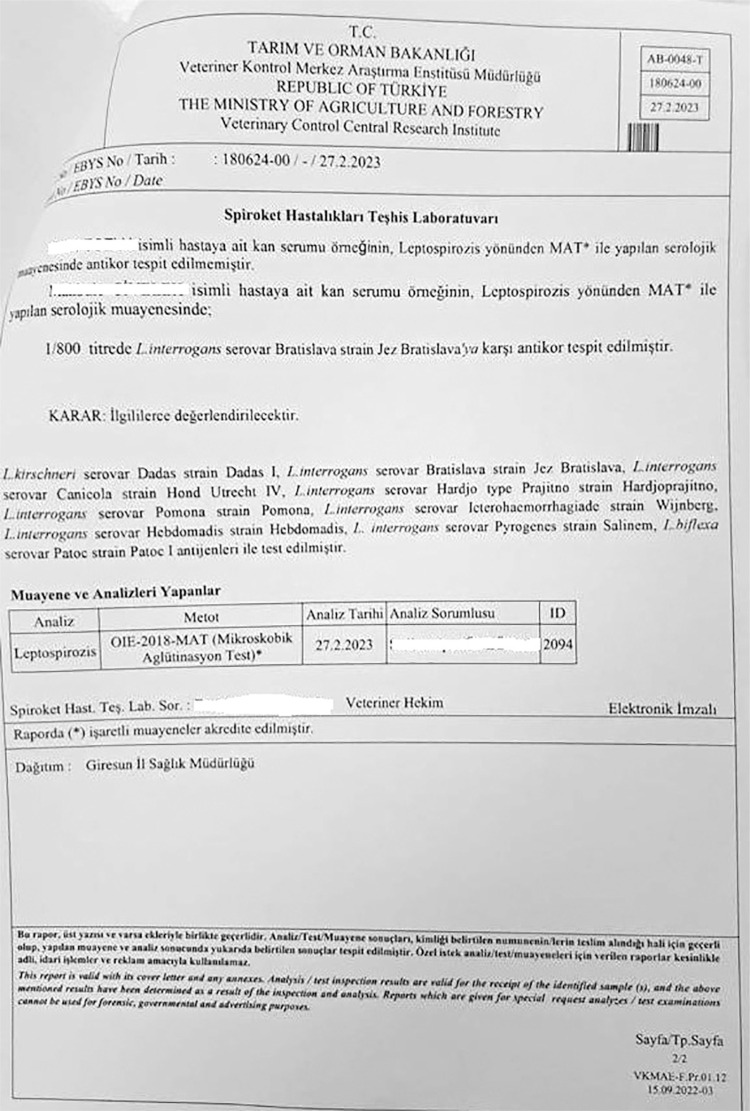
MAT result of the patient.

A more comprehensive anamnesis found that the patient who lives in the district center visited the village frequently and interacted with animals. The patient informed us that she had contact with chickens, cats, and dogs a week before the symptoms started. Serological tests for possible hantavirus infection and MAT test for possible *Leptospira* infection were requested for the patient under the coordination of Giresun Provincial Health Directorate. The results of the serological tests for hantavirus were negative. On the other hand, the *leptospira* MAT test performed at the Spirochet Diseases Diagnosis Laboratory of the Ministry of Agriculture and Forestry, Veterinary Control Central Research Institute revealed antibodies against *L. interrogans* serovar Bratislava type strain Jez Bratislava at 1/800 titer in the patient (Figure 1). Thus, a definitive diagnosis of leptospirosis was made. MAT was negative for other *Leptospira* serovars (Figure 1). Since ceftriaxone is one of the treatment choices in leptospirosis, and the patient’s clinical findings were improving with the help of supportive treatments, no additional therapy was applied. The patient was discharged on the fifth day of hospitalization.

## DISCUSSION

The studies conducted in Turkey demonstrated that leptospirosis seropositivity was higher in animals and humans living in coastal and rural areas^5^. Most studies on leptospirosis conducted in Turkey consist of case series and sporadic case reports. The case reports on leptospirosis were predominantly reported from the Black Sea, Mediterranean, and Marmara regions. Although Giresun is a city located in the Eastern part of the Black Sea region, now being the first in the region in which a case of LISB was reported. The first human case of LISB in the literature was reported in Italy in 1989^6^. Three cases of LISB were reported in the southern part of the Marmara region in 2009, and another case was reported in the central Anatolia region in 2005^7,8^. An epidemiological study comparing the prevalence of leptospirosis in people with risky jobs and the normal population conducted in the Eastern part of the Black Sea region revealed a significantly increased prevalence of leptospirosis on the former (farmers, paddy workers, animal keepers, land and fish hunters, veterinarians and veterinary technicians). The study reported only L.grippothyphosa and L.icterohaemorrhagiae infections in humans^9^. Studies worldwide also indicate the increased incidence of leptospirosis in patients who take part in occupational risk groups. For example, a recent study from Iran conducted with 279 subjects reported three positive cases of *Leptospira* infection, all in livestock farmers^10^. Our patient also had a history of animal contact. Therefore, patients with suspicious symptoms should be evaluated carefully, and the diagnosis of leptospirosis must be kept in mind, especially if it occurs in a occupational risk group.

Our patient had a mild/moderate clinical course, though some patients with leptospirosis may evolve to death. The clinical course of some patients might progress to severe hyperbilirubinemia, acute renal failure, hemorrhagic features, sepsis, multiple organ failure, coma, and death^11^. Due to this wide range of clinical spectrum, it might be challenging to make differential diagnosis from nonspecific diseases such as COVID-19^12^. Moreover, neither a routine laboratory test was reported as diagnostic nor was any association revealed between a lab test and mortality for this disease. Increased serum bilirubin, creatinine, and thrombocytopenia are the main lab features of the disease.^2^However, our case had markedly increased C-reactive protein, erythrocyte sedimentation rate, neutrophil count, and decreased lymphocytes without hyperbilirubinemia and thrombocytopenia. Thus, the diagnostic value of acute phase reactants and neutrophil/lymphocyte ratio should be studied. Supportive treatment with doxycycline, ampicillin, or ceftriaxone is usually sufficient and successful for treating mild/moderate forms of the disease. However, advanced therapies such as plasmapheresis, cyclophosphamide, and ventilatory support might be necessary for patients with severe leptospirosis^12,13^. Thus, all the suspected cases must be evaluated for the possible diagnosis of leptospirosis and, when diagnosed, carefully followed up for the severe disease. Fortunately, our patient had a mild form and healed with ceftriaxone plus supportive therapy.

## CONCLUSION

In conclusion, leptospirosis is a disease with nonspecific signs and symptoms and varying clinical courses. Clinicians should not overlook this diagnosis, especially in patients with unexplained fever, altered renal function tests, and acute phase reactants. This animal-borne infection should be taken seriously, and epidemiological studies with large human populations should be conducted since the studies in the literature are insufficient.
